# Lightweight Container Orchestration for Reproducible AI and Deep Learning Segmentation of Coronary Arteries and the Aorta in Coronary CT Angiography

**DOI:** 10.3390/diagnostics16142214

**Published:** 2026-07-15

**Authors:** Michal Iwanski, Piotr Regulski, Piotr Wendykier

**Affiliations:** Digital Imaging and Virtual Reality Laboratory, Department of Dental and Maxillofacial Radiology, Medical University of Warsaw, 02-097 Warsaw, Poland

**Keywords:** Podman, container orchestration, coronary CT angiography, segmentation, VisNow

## Abstract

**Background**: Deep learning segmentation of coronary CT angiography (CCTA) can support visualization, quantitative analysis, and patient-specific research workflows, but local deployment is often limited by the GPU configuration, dependency drift, heterogeneous operating systems, and limited DevOps resources. **Objective**: This study aimed to develop and evaluate a cross-platform orchestrator for the reproducible execution of containerized cardiovascular segmentation models without the need for Kubernetes. The segmentation models were used as representative demonstration workloads. **Materials and Methods**: A lightweight container orchestrator was developed using Podman and Podman Desktop Machine. The system provides a standardized REST API, asynchronous job execution, parallel and cascaded inference modes, persistent logging, and an external evaluation module. Two containerized nnU-Net v2 services were implemented for coronary artery and aortic segmentation from CCTA. Models were trained on the ImageCAS data and evaluated on an independent external cohort of 200 CCTA examinations. The performance was assessed using the Dice similarity coefficient (DSC) and intersection-over-union (IoU). System latency, throughput, and resource utilization were also measured. **Results**: The mean end-to-end latency on Linux was 52.3 ± 7.1 s with GPU acceleration and 74.1 ± 8.3 s in CPU fallback mode. Parallel execution increased throughput from 44.4 to 62.1 cases/hour, with the expected latency increase due to resource contention. As workload validation, the coronary and aortic nnU-Net services achieved DSC values of 0.93 and 0.95, respectively. **Conclusions**: The proposed orchestrator enables reproducible, standardized, portable deployment of containerized CCTA segmentation models in on-premises research environments, reducing operational barriers while supporting visualization, validation, and downstream applications.

## 1. Introduction

### Clinical and Educational Motivation

Coronary artery disease remains a leading cause of morbidity and mortality [1,2]. Coronary CT angiography (CCTA) is widely used to evaluate coronary anatomy and disease, and high-quality segmentation of coronary vessels and adjacent structures is a prerequisite for many downstream tasks: visualization, quantitative analysis, virtual modelling, and procedure planning [3,4].

High-quality segmentation of coronary arteries from CCTA plays a pivotal role in facilitating clinical interpretation by radiologists and interventional cardiologists. Automated delineation of the coronary tree enables clearer visualization of vessel course, branching patterns, and lesion localization, thereby supporting more efficient assessment of stenosis distribution, anatomical variants, and procedural feasibility [5,6]. By transforming complex volumetric datasets into structured anatomical representations, segmentation reduces the cognitive load and enhances the reproducibility of vessel-based measurements across observers [7]. These structured models can additionally serve as inputs for advanced visualization environments, including virtual reality-based procedural rehearsal systems. The development and validation of such segmentation approaches have been supported by publicly available datasets and benchmarks, which promote methodological transparency and objective comparisons across algorithms [8].

Although deep learning models, including self-configuring pipelines such as nnU-Net, can achieve strong segmentation performance, their deployment in clinical practice is often impeded by practical constraints [9]. These include limited or heterogeneous computational infrastructure (such as GPU availability, memory capacity, and dependency on CUDA drivers and specialized numerical libraries), as well as restricted IT and DevOps support within clinical or academic teams [10]. In addition, fragmentation across software frameworks, data formats, and evaluation protocols hampers standardization and complicates fair comparisons between methods [11]. Finally, reproducibility can be undermined by environmental drift and inconsistent software stacks, making it difficult to replicate results reliably across sites and over time.

Existing production-oriented approaches often assume the availability of Kubernetes, which is usually infeasible for smaller research groups and mixed Windows/Linux environments.

The aim of this study was to develop a lightweight, container-based AI model orchestrator that standardizes inference execution, supports both sequential (cascaded) and parallel processing pipelines, and operates in a cross-platform environment (Linux, Windows) without requiring a full-scale cluster management solution. The proposed system was designed to lower infrastructural barriers while ensuring the reproducibility, modularity, and extensibility of deployed CCTA segmentation models in an on-premises research setting.

## 2. Materials and Methods

### 2.1. Orchestrator Architecture and API Surface

The orchestrator was implemented as a modular “platform-in-the-middle” layer positioned between client applications (visualization tools, research interfaces, and automated workflows) and containerized AI inference services. Architecturally, the system comprises an application programming interface (API) layer, a scheduling and execution engine, a model registry, a storage and logging subsystem, and an evaluation module connected through explicit internal interfaces to maintain the separability of concerns and facilitate maintainability.

The orchestrator exposes a unified REST API for external client applications. In the implemented system, external clients do not call model containers directly. A new asynchronous inference task is created through POST/inference, which returns a unique job_id. Job progress is monitored through GET/status/{job_id}, and completed output artefacts are retrieved through GET/results/{job_id}. Model discovery is provided through GET/models and GET/models/{model_id}, while service availability is checked through GET/health.

**Figure 1:**
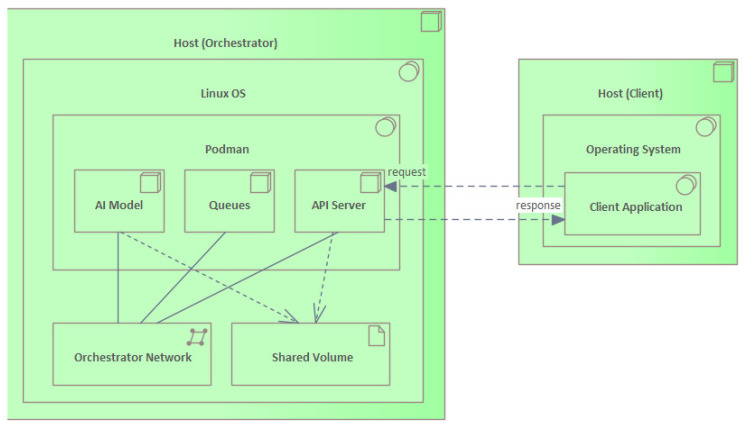
Technology layer architecture. A technology-based layer view was developed using the ArchiMate modelling language. The diagram presents the underlying infrastructure, host environment, system software components, and data flows between systems. It highlights the deployment perspective of the proposed solution, including the distribution of software components across one or more hosts, in accordance with established Enterprise Architecture principles and the official ArchiMate specification.

Internally, the orchestrator invokes individual model-runner containers through a separate standardized model interface. This internal interface includes POST/predict for model inference, GET/health for readiness checks, and GET/metadata for model-specific information. This API design enforces a stable, versioned contract that decouples client implementations from model-specific details so that changes in model architecture, framework dependencies, or container runtime stacks do not propagate to the client layer, provided that the defined input–output schema is preserved (see Figure 1).

### 2.2. Model Registry, Container Execution, and Standardized Model Interface

A central component of the orchestration layer is the model registry, which maintains the structured metadata required for deterministic execution and reproducible benchmarking. Each registry entry includes a model identifier, semantic version, container image reference, declared input and output formats, resource requirements (including GPU dependency), and optional descriptive metadata. This registry supports dynamic discovery at runtime and the controlled coexistence of multiple model versions, enabling longitudinal comparisons and reproducible re-execution. Model weights are not stored in the registry. Instead, they are encapsulated within versioned container images to keep runtime environments tightly coupled to specific model versions.

Each model is packaged as an isolated container image that encapsulates the complete inference environment, including framework libraries, numerical dependencies, and trained weights. The orchestrator launches containers using a daemonless runtime, ensuring process isolation and minimizing cross-model interference. During execution, the input data are mounted and transferred into the container context, and the output artefacts are retrieved upon completion. This isolation eliminates dependency conflicts and reduces environmental drift while limiting access to well-defined filesystems and runtime boundaries.

To ensure uniform interoperability, every model container is required to implement an internal standardized service contract used by the orchestrator. Specifically, model runners expose POST/predict for inference execution, GET/health for readiness/liveness reporting, and GET/metadata for structured introspection (model name, version, task type, expected input, output schema, and hardware requirements). Input payloads are supported as volumetric medical imaging data (NIfTI volumes or DICOM series), and outputs are returned as segmentation label maps (NIfTI masks) optionally accompanied by minimal structured metadata required for traceability. Enforcing this common contract allows for the integration of models implemented in heterogeneous frameworks (PyTorch v2.4.0, TensorFlow v.2.10.0 runtimes) while maintaining consistent invocation semantics and evaluation pathways.

The orchestrator follows an asynchronous execution model in which inference requests are registered by the API gateway and delegated to the scheduling and execution engine. The orchestrator supervises job lifecycle states (registered, running, completed) and coordinates container invocation accordingly. Two execution paradigms are supported: parallel mode, in which multiple models process the same input independently, and cascaded mode, in which the output of one model becomes the input of a subsequent model. Resource management is handled at execution time on the basis of registry-declared requirements. GPU-accelerated execution is enabled when resources are available, while CPU execution remains supported to preserve portability across heterogeneous institutional infrastructures.

Incoming inference requests are assigned a unique job_id, stored with metadata, and dispatched asynchronously through Redis/RQ queues (v5.0.0). In the present implementation, queue selection is based on the requested model and priority level. Separate queues are maintained for supported models and for normal/high priority execution, allowing workers to process jobs asynchronously while preserving a lightweight single-host orchestration model (see Algorithm 1).

For single-model inference, the uploaded input file and metadata are validated, the target model is resolved, and the corresponding model function is enqueued as an RQ job. Parallel execution is achieved by submitting independent jobs, which may be processed concurrently when multiple workers and sufficient local resources are available. For cascaded inference, the pipeline endpoint enqueues a sequence of dependent jobs using RQ depends_on. Each downstream step is released only after successful completion of the previous step and uses the upstream output as its input. If a pipeline step fails, queued dependent steps are cancelled.

Resource handling is performed at the worker/container deployment level. GPU-accelerated or CPU execution is therefore determined by the configured worker, container image, and host or Podman-machine environment, while the orchestrator provides standardized queuing, job tracking, status reporting, and result retrieval.
**Algorithm 1.** Pseudocode of the simplified orchestration logic.Input: POST request containing an uploaded imaging file and JSON metadataOutput: job_id or pipeline_id, job status, and output artefacts1. Receive request through POST/inference or POST/pipeline2. Validate metadata and save the uploaded input file3. If the request is a single-model inference: {4.    Read Model and Priority from metadata5.    Select the RQ queue using (Model, Priority)6.    Enqueue the corresponding model function as an asynchronous job7.    Return job_id, queue name, priority, and initial status}8. If the request is a multi-step pipeline: {9.    Create pipeline_id10.   For each pipeline step:11.     Read Model and Priority12.     Select the RQ queue using (Model, Priority)13.     Enqueue the step as an asynchronous job14.     If this is not the first step, set depends on to the previous job15.   Store pipeline_id and all job_ids in Redis16.   Return pipeline_id and step job_ids}17. RQ workers execute queued jobs asynchronously18. Status endpoints read job or pipeline state from Redis/RQ19. Result endpoints return the completed job result or the final pipeline output20. If a pipeline step fails, queued dependent steps are cancelled

The infrastructural layer is based on Podman and Podman Desktop Machine v5.8.5 (PDM). Containers execute natively on Linux hosts and via lightweight Linux virtual machines on non-Linux hosts, providing consistent behaviour across platforms without the need for Kubernetes or external cluster services.

### 2.3. Demonstration AI Workloads and Datasets

To instantiate and test the orchestrator with realistic medical imaging workloads, two independent segmentation services based on nnU-Net v2 were selected as a robust, self-configuring framework that reduces engineering overhead while providing reproducible, high-performing baselines for medical image segmentation. The first model targets coronary artery lumen and tree segmentation, and the second model targets aortic segmentation as a single anatomical class. Both services operate on the same CCTA input and, in the presented workflow, are executed in parallel mode. Cascaded execution remains available for scenarios requiring multistage processing but was not required for the coronary/aorta segmentation tasks as implemented.

Both segmentation models were configured in a 3D full-resolution setup appropriate for volumetric CCTA data. Preprocessing standardized the input geometry by resampling to an isotropic voxel spacing of 0.5 mm and *CTNormalization* based on foreground intensity statistics. The *3d_fullres* model used a *PlainConvUNet* architecture with a patch size of 96 × 160 × 160 voxels, a planned batch size of 12, 32 base features, 320 maximum features, and 3 × 3 × 3 convolution kernels. Training followed the standard nnU-Net v2 configuration with Dice-plus cross-entropy loss, deep supervision, SGD with Nesterov momentum, polynomial learning-rate decay, and 1000 training epochs. Post-processing was limited to connected-component filtering to remove isolated artefacts and improve anatomical plausibility. For downstream geometry-based use, segmentation masks were converted automatically to surface meshes (STL) with minimal smoothing to preserve anatomical fidelity. Inference was executed within the containerized environment with GPU acceleration when available and CPU fallback otherwise.

Both the coronary segmentation model and the aorta segmentation model were trained using the publicly available ImageCAS dataset, comprising 810 contrast-enhanced CCTA examinations and 190 cases reserved for validation as defined by the dataset protocol [12]. In the original ImageCAS annotation protocol, the left and right coronary arteries in each CTA volume were independently labelled by two radiologists and cross-validated; in cases of discrepancy, a third radiologist performed the annotation, and the final reference label was determined by consensus [12]. Because the original ImageCAS labels were designed for coronary artery segmentation, manual aortic masks were additionally generated in-house on the ImageCAS CCTA volumes by two expert readers—a radiologist with 15 years of experience and a cardiologist with 5 years of experience—and the final reference label was determined by consensus.

The final performance was evaluated on an independent external test cohort of 200 CCTA examinations acquired at the First Chair and Department of Cardiology, Medical University of Warsaw, consisting of consecutive adult patients who underwent clinically indicated CCTA prior to any coronary intervention. The mean age at CCTA was 64.5 ± 11.3 years, with a median of 65.7 years [IQR, 58.3–72.7] and a range of 33.0–90.8 years. Among the examinations, 49.5% were male, and 50.5% were female. CCTA was performed using a Toshiba Aquilion ONE CT scanner (Canon Medical Systems, Otawara, Japan), with a tube voltage of 135 kVp, a tube current of 660 mA, and a slice thickness of 0.5 mm. The images were stored in DICOM format; the previously reported median image matrix was 512 × 512 × 560 voxels, with a median voxel size of 0.43 × 0.43 × 0.5 mm [2]. Coronary artery annotations were performed on source CCTA volumes in VisNow by a cardiologist with 5 years of experience and independently reviewed by an experienced radiologist with 15 years of experience, following the consensus-based workflow. Discrepancies were resolved by consensus. For aortic segmentation, reference masks were generated and reviewed using the same expert-consensus principle. Inter-rater agreement for aortic mask delineation, calculated as the Dice similarity coefficient between the two independent pre-consensus annotations, was 0.96, indicating excellent agreement between the two readers. Readers were blinded to the automatic segmentations during reference mask generation and review. The protocol was approved by the local ethics committee (AKBE/245/2022).

This external testing setup ensured institutional and acquisition separation from the training data. Across all the datasets, partitioning was performed at the patient level to prevent data leakage, and no images from the independent test cohort were used for training, validation, or hyperparameter optimization.

### 2.4. Client Integration and End-to-End Workflow

**Figure 2:**
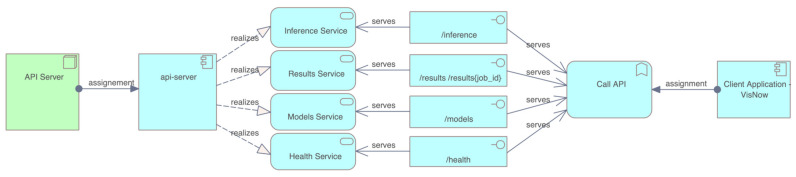
Application layer architecture. Application-layer view developed using the ArchiMate modelling language. The diagram illustrates the internal structure of the orchestrator and its relationships with other systems, including the interfaces and services responsible for receiving, managing, and executing inference requests to AI models. The model emphasizes the internal software complexity hidden from the end user and supports semantic clarity, reproducibility, and comparability with other studies based on the same modelling standard.

VisNow software v1.4.0 (Scientific Visualization Society—VisNow.org, Warsaw, Poland) served as the client-side interface, interacting with the orchestrator via the standardized API. VisNow is a modular, dataflow-oriented visualization platform implemented in Java and run using OpenJDK 17, enabling the construction of interactive processing pipelines using composable modules [13,14]. In this system, VisNow does not execute inference locally. Instead, it acts as an external API client responsible for data ingestion, task submission, result inspection, and artefact export (see Figure 2).

A dedicated VisNow module implements the operational workflow. The user loads a CCTA study in DICOM or NIfTI format and submits it to the orchestrator through the external inference endpoint, POST/inference. The orchestrator registers the request as an asynchronous job and returns a job_id. VisNow then monitors the job progress through GET/status/{job_id} until the job is completed and retrieves the generated artefacts through GET/results/{job_id}. The /predict endpoint is used only internally by the orchestrator when the selected model-runner container is invoked. Returned segmentation masks are visualized as volumetric overlays and three-dimensional surface reconstructions to support immediate quality control. After inspection, the NIfTI masks and postisosurface STL meshes are exported for downstream use. In the present implementation, one downstream consumer was a VR angioplasty simulation pipeline.

### 2.5. Evaluation Protocol

Evaluation was performed at two complementary levels. The primary evaluation target was system-level deployment behaviour, including successful cross-platform execution, standardized invocation, latency, throughput, resource utilization, logging, and result retrieval. Segmentation quality metrics were retained as validation of the demonstration workloads and as a test of the orchestrator-side evaluation module. The Dice similarity coefficient (DSC) was the main workload-quality metric. In addition, the evaluation component supports standardized overlap- and surface-based metrics, including intersection-over-union (IoU), to facilitate benchmarking across the models registered in the system. Metric computation is implemented outside the model containers to prevent discrepancies due to heterogeneous in-container evaluation scripts.

For system-level performance, inference latency was measured as the wall-clock time from external job submission through POST/inference to output availability through GET/results/{job_id}. This interval included orchestrator handling, internal model invocation, inference, postprocessing, and artefact packaging. Measurements were repeated to quantify variability and were summarized using the mean and standard deviation. Each metric was computed from 2 repeated runs per case after an initial warm-up of 2 runs to mitigate cold-start effects.

All the experiments were performed on the following reference hardware and operating environments: CPU: Intel Core i5-13600HX (cores/threads: 14/20; base/turbo: 2.60/4.80 GHz), RAM: 64 GB, storage: 1024 GB (NVMe SSD), and GPU: NVIDIA RTX 3500 Ada Generation Laptop GPU (VRAM: 12 GB; driver: 537.13; CUDA: 12.2). The orchestrator stack was executed in two deployment configurations: (i) native Linux execution using Podman (OS: Linux Mint 22 (Wilma); kernel: 6.8.0–51-generic; Podman: 3.4.2; OCI runtime: crun; filesystem backend: overlay); and (ii) Windows execution using a Podman Desktop podman-machine Linux VM (Windows: 10; podman-machine: podman-cardio-machine; VM backend: WSL kernel; VM resources: CPU: 16, RAM: 2048 MB; guest OS: Fedora Linux). The orchestrator version was fixed to 1.0 (release/tag/commit). Runtime parameters that influence performance were explicitly recorded, including the container execution mode (rootless/rootful: rootless), GPU enablement flags (WSL GPU passthrough), any CPU threading settings, and any container-level resource limits inside the Podman virtual machine.

To further characterize runtime behaviour, the end-to-end execution time was decomposed into preprocessing, model inference, postprocessing, input/output handling, and container-related overhead. Timing was performed using timestamped instrumentation around each pipeline stage. Input/output time included saving the uploaded input file, passing artefacts between the orchestrator and model service, and registering the output files for retrieval. Container-related overhead included container/API hand-off and invocation costs outside the model computation. Model inference time was calculated as the residual component after subtracting measured non-inference overheads from the end-to-end latency.

Parallel execution behaviour was assessed by submitting multiple concurrent requests and analysing the response time on native Linux throughput, as well as resource contention effects. Hardware resource consumption was monitored during inference, including GPU memory utilization (when acceleration was enabled), CPU memory usage, and peak RAM consumption. Runtime stability was assessed through repeated functional executions under the same container image versions and runtime configurations, with emphasis on successful job completion, result retrieval, and the absence of container-level execution failures.

To assess the computational impact of input size on nnU-Net execution time, the input size in kilobytes and the corresponding inference time for each case were recorded. The relationship between input size and inference time was assessed using Pearson and Spearman correlation coefficients, as well as simple linear regression.

## 3. Results

Results are presented with the orchestration system as the primary contribution. The orchestrator was successfully instantiated as a complete, modular service stack comprising an API gateway, model registry, containerized model runner services, persistent storage/logging, and a standalone evaluation component. Container execution was validated on Linux hosts using native Podman and Windows hosts via a podman-machine Linux virtual machine, which demonstrated that the same model containers can be executed in heterogeneous on-premises environments without a cluster-level orchestrator. The reference nnU-Net container implemented the standardized model-serving contract required by the orchestrator, exposing /predict for inference execution, /health for operational readiness checks, and /metadata for structured model introspection, which enabled uniform discovery, invocation, and monitoring of services through a consistent API surface.

**Figure 3:**
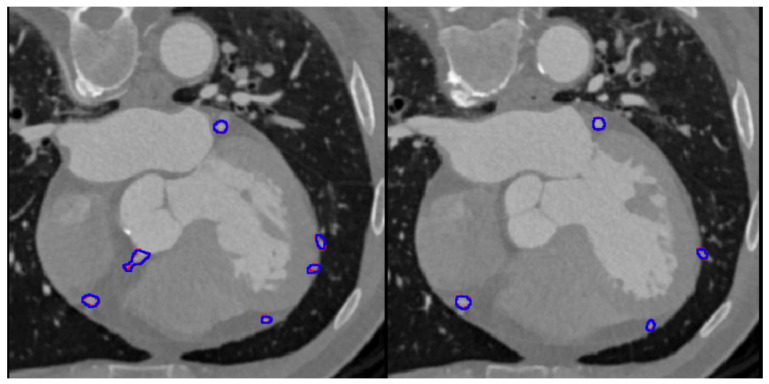
Manual (blue) vs. automatic (red) outline of segmented coronary arteries.

As validation of the demonstration workloads, in the independent external test cohort (*n* = 200), the containerized nnU-Net coronary artery segmentation service achieved a mean Dice similarity coefficient (DSC) of 0.93 (95% CI: 0.925–0.934), with a median DSC of 0.933 (95% bootstrap CI: 0.931–0.935), standard deviation of 0.036, and range of 0.755–0.988. The interquartile range was 0.910–0.954. Per-case analysis showed that 71.1% of cases achieved DSC ≥ 0.90, while 4.0% of cases had DSC < 0.80. The corresponding overlap, expressed as intersection-over-union, was IoU = 0.87 for a binary mask (see Figure 3).

**Figure 4:**
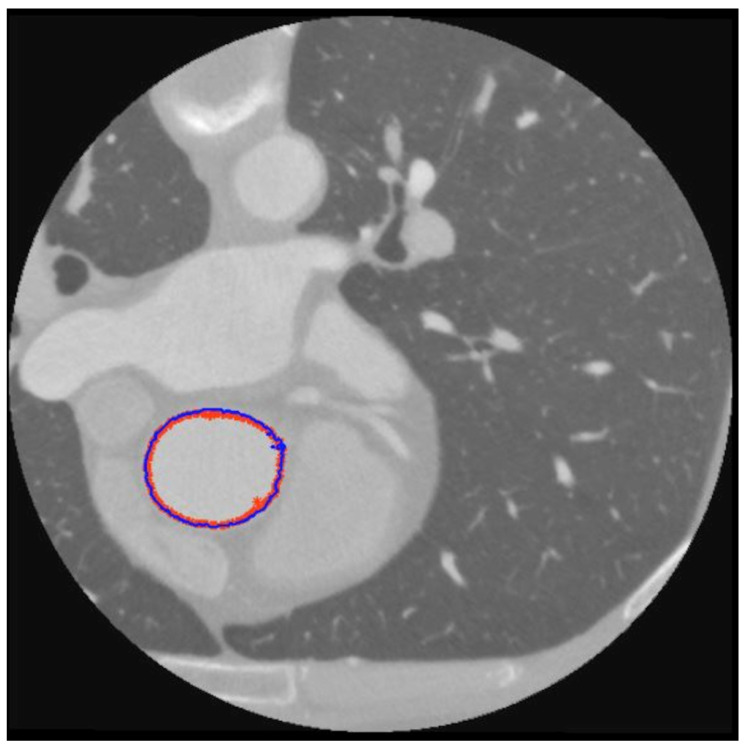
Manual (blue) vs. automatic (red) outline of the segmented aorta.

The aorta segmentation service likewise demonstrated high agreement with the expert-consensus reference masks, achieving a mean DSC = 0.95 (95% CI: 0.905–0.981), with a median DSC of 0.985 (95% bootstrap CI: 0.982–0.992), a standard deviation of 0.018, and a range of 0.855–0.988. The interquartile range was 0.941–0.989. Per-case analysis showed that 90% of cases achieved DSC ≥ 0.90. These results correspond to an IoU = 0.90. All segmentation metrics were computed by the orchestrator-side evaluation module operating outside the model containers, ensuring that identical metric implementations and parameterizations were applied across model versions and deployments, independent of framework-specific evaluation code packaged within individual containers, and enabling direct benchmarking of registered segmentation services without container modification (see Figure 4).

Failure-case screening identified 4% low-DSC outliers using the IQR criterion (DSC < 0.8). Quantitative inspection suggested two main error patterns: false-positive (FP)-dominant cases consistent with over-segmentation/leakage into adjacent structures and false-negative-dominant cases consistent with under-segmentation or missed anatomical portions. The lowest-performing case had DSC = 0.755 in coronary artery segmentation, indicating FP-dominant segmentation.

System-level performance was quantified as end-to-end wall-clock latency measured from external API job submission through POST/inference to the point at which all output artefacts were available for retrieval through GET/results/{job_id}. For single-service inference on native Podman Linux, the mean end-to-end latency was 52.3 ± 7.1 s with GPU acceleration and 74.1 ± 8.3 s in CPU fallback mode, with estimated P95 values of 64.0 s and 87.8 s, respectively. For Windows using PDM, the mean end-to-end latency was 59.4 ± 12.1 s with GPU acceleration and 86.6 ± 11.9 s in CPU fallback mode. The corresponding P95 values were 79.3 s and 106.2 s, respectively. These results indicate that the proposed orchestration layer enables practical execution times for interactive research and visualization workflows while preserving container isolation and standardized model invocation.

A detailed timing decomposition was performed. The measured non-inference overhead was small relative to the total execution time. Preprocessing required 1 ± 1 ms, postprocessing required 15 ± 4 ms, input/output handling required 39 ± 16 ms, and container-related overhead required 55 ± 18 ms. Together, these non-inference components accounted for approximately 0.11 s of the 52.3 ± 7.1 s end-to-end latency. Thus, the execution time was dominated by model inference, which accounted for approximately 52.19 ± 7.10 s.

Parallel execution behaviour was assessed on native Podman Linux by submitting multiple concurrent requests and by invoking the coronary artery and aorta segmentation services in parallel on the same input study data. Under single-request execution, the throughput was 44.4 cases/hour. Under a parallel load of 2 concurrent jobs, the throughput increased to 62.1 cases/hour, while the median end-to-end latency increased from 81.0 s to 116.0 s and the P95 latency increased from 92.4 s to 133.5 s, reflecting the expected resource contention. When both segmentation services were executed concurrently for the same study data, the combined per-case end-to-end time, defined as the time until both outputs were available, was 116.0 s on the GPU and 139.0 s on the CPU. Hardware utilization measurements revealed a peak GPU memory footprint of 6.4 GB for coronary segmentation and 4.2 GB for aorta segmentation, with a combined peak of 10.2 GB during parallel execution; the peak host RAM consumption was 24.6 GB, and the peak CPU memory attributed to the container runtime and orchestrator processes was 3.4 GB. No sustained memory growth across repeated runs was observed, with a peak-to-peak drift of 2.1% across consecutive executions, indicating stable resource behaviour under the tested workloads.

A positive association was observed between input size and inference time, with Pearson’s r = 0.713 (*p* = 0.021) and Spearman’s ρ = 0.794 (*p* = 0.006). Linear regression indicated an average increase of approximately 15.0 s per additional 10,000 KB of input data, with input size explaining 50.9% of the variability in execution time.

From the client perspective, VisNow enabled a concise, few-step workflow that operationalized the end-to-end inference pipeline within a single modular environment: study import (DICOM/NIfTI), API-based invocation of the containerized services through the orchestrator, automated retrieval of completed results, interactive inspection of segmentation overlays and 3D surface reconstructions, and export of output artefacts as STL meshes (and, where needed, volumetric masks). This integration aligns with VisNow’s dataflow-driven philosophy and plugin architecture, allowing for the inference call, visualization, and export operations to be composed into a reusable processing graph. The exported STL surfaces were subsequently important as patient-specific assets in downstream tooling, including the VR angioplasty simulation pipeline used in this project.

## 4. Discussion

The present study demonstrates that a lightweight, Podman-based orchestration layer can provide standardized, reproducible AI inference in settings where Kubernetes-centric tooling is difficult to deploy or maintain. The main contribution is the orchestration approach: a cross-platform API-based execution layer with a model registry, isolated container runtime, asynchronous job handling, parallel workflow support, logging, and centralized evaluation. By positioning the orchestrator as “platform-in-the-middle” between client applications and containerized inference services, the approach enables end-to-end pipelines that remain operational on workstations and on-premises servers, which is a practical deployment class for many clinical research groups. In this context, containerized nnU-Net services provide a strong and methodologically well-justified baseline for coronary and aortic segmentation, combining high segmentation performance with a reduced engineering burden because of the self-configuring design of nnU-Net. Importantly, integration with VisNow provides a clinically meaningful interaction model and allows for visualization, validation, export, and optional simulation downstream, implemented within a modular, dataflow-driven paradigm that supports rapid construction of domain-specific pipelines through plugins and composable modules [13,14].

A central contribution of this work is the explicit trade-off between “industrial-scale” orchestration and deployability under institutional constraints. Kubernetes-based stacks (including Kubeflow ecosystems and Kubernetes-native serving frameworks) are well suited for multitenant, autoscaled production deployments, but they impose nontrivial operational complexity, including cluster provisioning, security hardening, and ongoing DevOps maintenance [15,16]. In contrast, the proposed orchestrator deliberately avoids operating as a cluster manager. Instead, it implements only the minimal control plane required to standardize execution and evaluation across model containers. This design choice lowers the barrier to adoption in environments that lack dedicated platform teams while retaining architecture-level rigor through a stable API contract, a model registry with explicit versioning, and centralized, uniform metric computation. From a methodological perspective, the key implication is that reproducible inference and benchmarking do not strictly require the full complexity of Kubernetes if the primary need is the deterministic, auditable execution of containerized models on limited local infrastructure [17].

Podman was selected to minimize the DevOps and system-administration knowledge required to run the tool. The containerization layer therefore had to remain simple, self-contained, low-maintenance, and independent of a privileged background service. In typical deployments, Docker relies on a continuously running dockerd daemon with root privileges and exposes a socket that can provide root-equivalent access, increasing the attack surface in single-host environments without dedicated security teams [17]. The daemon also represents a single point of failure: if it crashes or fails to start, containers become unmanageable despite the workload not requiring centralized orchestration. Lightweight Kubernetes distributions were also not selected because they are designed for workload orchestration, scheduling, service discovery, self-healing, and cluster-state management and still require familiarity with pods, manifests, controllers, kubelet, and clustered networking. This conflicted with the goal of minimizing operational complexity. Podman instead launches containers as regular child processes of the invoking user, requires no background daemon, supports direct command-line interface management, and can also be integrated with system services. Its rootless model, based on Linux user namespaces, reduces security exposure without requiring additional operator expertise. Podman can also use the system for container management via Quadlet. For these reasons, Podman was considered appropriate for the requirements of this project: it removes the need for a system daemon, supports rootless execution, reduces administrative overhead, and is therefore technically and organizationally justified as the containerization tool for this workflow.

A related comparison arises with ML lifecycle tooling, such as MLflow. MLflow formalizes core lifecycle needs, experimental tracking, model packaging, and model registry/versioning, highlighting the importance of lineage and reproducibility in practical ML systems [18]. However, in many clinical research deployments, the “last mile” challenge is not only model tracking but also operationalizing inference execution across heterogeneous machines and software stacks with minimal IT support. In this context, the orchestrator complements ML lifecycle tooling by focusing on standardized inference execution and evaluation under container isolation rather than attempting to provide a comprehensive end-to-end training and continuous integration stack [19]. Conceptually, the orchestrator can be viewed as a pragmatic inference substrate that is compatible with the principles promoted by MLflow (versioned artefacts, traceability) but optimized for environments where cluster-level scaling assumptions are not realistic [19].

When situated within the broader landscape of containerized medical AI deployment frameworks, the proposed approach is aligned with a growing emphasis on packaging, portability, and workflow composability. MONAI Deploy, for example, operationalizes medical imaging inference via operator graphs and infrastructure integration (including clinical imaging workflows such as PACS connectivity), representing an increasingly mature path towards the research and clinical deployment of AI workflows [20]. Similarly, platforms such as Kaapana aim to provide an integrated, end-to-end research environment for medical imaging, covering ingestion, analysis workflows, and collaboration at a larger platform scope [21]. In contrast, the present work targets a narrower but practically important gap: providing a lightweight orchestration mechanism for inference execution and benchmarking that can be deployed without the institutional prerequisites of a full platform stack while remaining compatible with downstream integration patterns.

The relevance of containerization to vessel-oriented segmentation workflows is further supported by multiple strands of prior work that explicitly adopt container-based distribution or evaluation. CardioVision presents a fully automated deep learning package for segmentation and reconstruction in the context of aortic stenosis “digital twins,” illustrating how packaging a complex pipeline improves usability and facilitates dissemination [22]. The SEG.A 2023 challenge, focused on the automatic segmentation, modelling, and meshing of aortic vessel trees, explicitly adopted a “container submission” paradigm in which participants submitted algorithms as Docker containers to ensure standardized execution and evaluation [23].

Tools such as TotalSegmentator further reflect the field’s direction towards robust, broadly applicable segmentation toolkits that can be distributed in a way that reduces dependency friction and supports consistent execution across sites [24]. The present study advances existing container-based approaches by formalizing orchestration as a standardized execution layer rather than merely a packaging mechanism. Specifically, multiple vessel-related segmentation services (coronary arteries and aorta) are executed under a unified model registry, a consistent API contract, and a centralized evaluation framework. This design supports both parallel execution of independent tasks and cascaded workflows for modular composition while maintaining version control and reproducibility across deployments (see Table 1).

Containerization is particularly consequential for reproducibility because it addresses a common failure mode in translational AI-environment drift and nonportable software stacks [26]. Prior work in biomedical image processing has emphasized that container-based pipelines improve reliability by copackaging dependencies, standardizing execution, and reducing discrepancies between development and deployment environments [27,28]. The present architecture builds on this principle by computing evaluation metrics outside model containers, thereby eliminating a subtle but important source of irreproducibility: heterogeneous “in-container” evaluation scripts that may differ across model implementations. Additionally, adherence to open container standards supports portability: the Open Container Initiative provides open specifications for container formats and runtimes, enabling a model packaged once to be executed across compliant runtimes and platforms [29,30]. In this work, Podman is used as the container runtime, offering a daemonless execution model and supporting operation without requiring privileged, always-on background daemons, which can be advantageous in restricted institutional environments.

Several limitations should be noted. First, while the segmentation services achieve high quantitative performance, generalizability remains constrained by dataset composition, annotation variability, and the intrinsic difficulty of coronary segmentation, particularly for small-calibre distal branches and regions affected by limited contrast opacification. CCTA is also susceptible to motion artefacts and calcification-related stenoses, both of which can degrade segmentation fidelity and are widely recognized as sources of failure in automated analysis. Such heterogeneity can induce case-specific errors that are not fully captured by aggregate overlap metrics.

Second, the non-Linux execution pathway (via a Podman-managed virtual machine on hosts such as Windows) introduces additional system complexity and may add measurable overhead. In practice, GPU pass-through and driver alignment can be constrained by local hardware and institutional IT policies, which may limit acceleration in some installations. In the present implementation, GPU acceleration and CPU fallback were evaluated within the same Podman-based workflow, whereas FPGA execution was not implemented. From a containerization perspective, CPU execution is the lowest-friction fallback because standard OCI images run without device passthrough, special drivers, or vendor-specific runtime configuration. GPU execution was selected as the primary inference target because it provided lower per-case latency in our measurements and has mature container support. NVIDIA GPUs can be exposed to rootless Podman through the Container Device Interface, which remains consistent with the daemonless design of the system. In contrast, FPGA deployment typically depends on vendor-specific stacks such as AMD/Xilinx Vitis AI or Intel/OpenVINO-based FPGA workflows, large vendor images, manual device-node passthrough, board-specific bitstream provisioning, and often root or privileged container execution. These requirements conflict with the intended low-DevOps, rootless deployment model. Therefore, GPU was used as the primary inference target, CPU was retained as a zero-passthrough fallback for environments without GPU access, and FPGA support was left for future work requiring dedicated hardware, energy measurements, and specialized FPGA engineering expertise.

Third, the lightweight design intentionally deprioritizes features typically handled by Kubernetes ecosystems (autoscaling, multitenant governance, and service meshes); therefore, the approach is best interpreted as complementary to enterprise deployment stacks.

Fourth, the system was evaluated as an on-premises research workflow and should not yet be considered a clinical deployment solution. Although the orchestrator supports standardized inference execution and VisNow-based inspection, several requirements for routine clinical integration remain outside the scope of this study, including PACS/DICOMweb connectivity, user authentication, role-based authorization, audit logging, cybersecurity assessment, regulatory compliance analysis, and prospective evaluation in real clinical decision-making workflows. These aspects are planned as future extensions.

Future work will focus on expanding the ecosystem of containerized services and increasing the degree of clinical workflow alignment. Methodologically, the orchestrator is naturally suited to hosting additional containers for stenosis detection, plaque characterization, centreline extraction, vessel straightening, and potentially flow-related analyses, enabling modular pipelines that remain versioned, auditable, and benchmarkable under a unified evaluation layer. From an integration perspective, deeper connectivity to clinical infrastructure, such as PACS ingestion, DICOM segmentation export, and stronger audit logging and governance controls, would move the system towards more routine deployment scenarios and align with evolving medical ML operations practices. Finally, multicentre evaluation and, where feasible, federated learning or cross-site benchmarking would provide stronger evidence for robustness under real-world heterogeneity and would better quantify the practical deployment advantages of standardized, container-based orchestration in cardiovascular imaging workflows.

## 5. Conclusions

We propose a cross-platform, lightweight container orchestration approach for medical AI inference based on Podman and PDM, which is designed to lower operational barriers and increase reproducibility for AI-driven models. The primary contribution is the orchestration layer, which standardizes model invocation, execution, logging, evaluation, and result retrieval across heterogeneous on-premises environments without requiring Kubernetes. The feasibility of the approach is demonstrated through a containerized nnU-Net model for coronary artery and aorta segmentation and an interactive VisNow client workflow that supports visualization, validation, and export of results for downstream applications, including VR angioplasty simulation.

## Figures and Tables

**Table 1:** Containerized deep learning frameworks for aortic and coronary CT segmentation: comparison of platforms, content, and reported performance.

Author	Container	Content	Usability	Measures
This work	Podman/PDM	Containerized nnU-Net segmentation services for CCTA (coronary artery lumen/tree and aorta) segmentation	Lightweight, Kubernetes-free orchestration; cross-platform execution (Linux/Windows); support for parallel and cascaded execution; on-premises deployment; reproducible inference and benchmarking via registry-based model versioning	Coronary segmentation: DSC 0.93, IoU 0.87 Aorta segmentation: DSC 0.95, IoU 0.90
Rouhollahi et al. [22]	Docker	Python pipeline + DCNN module for aortic segmentation (aortic root/ ascending aorta segments)	Containerization as a cross-platform layer and environment standardization (Windows/Linux/macOS) for a packaged aortic segmentation pipeline	IoU 95.6%, DSC 97.8%, (for aorta segmentation)
Pepe et al. [23]	Docker	Collection of methods using various segmentation architectures for aortic vessel tree segmentation, modelling, and meshing	Container as a comparative benchmark artefact, ensuring result comparability	Phase 1 (mean): DSC 0.836–0.942, Phase 2 (mean): DSC 0.816–0.920 (for aorta segmentation)
Wasserthal et al. [24]	Docker	Segmentation model(s) (nnU-Net-based) + full inference dependencies; multiorgan CT segmentation including the aorta	Containerization as convenient inference distribution and mitigation of dependency drift; easy execution across environments	Mean performance across 104 anatomical structures: mean DSC 0.943 (95% CI 0.938–0.947)
Tölle et al. [25]	Docker (orchestration with Kaapana)	Application/pipeline containers executed locally at participating centres; includes CCTA segmentation tasks (including a coronary component as downstream task)	Containers as deployment units for federated learning/evaluation, providing isolation and environment standardization across institutions	coronary artery segmentation (ImageCAS) SWIN-UNETR Dice 0.683, UNet Dice 0.410; IoU SWIN-UNETR 0.140, IoU UNet 0.023

## Data Availability

The ImageCAS dataset is publicly available from the original dataset source. The independent external CCTA cohort cannot be made publicly available because of patient privacy and institutional restrictions. The aggregated evaluation results are available from the corresponding author upon reasonable request. The VisNow client source code is available from https://gitlab.com/visnow.org/VisNow (accessed on 25 May 2026). Orchestrator source code is available from https://github.com/cardioderm-ai/Cardio-Orchestrator (accessed on 25 May 2026).
